# Building resilience to mosquito-borne diseases in the Caribbean

**DOI:** 10.1371/journal.pbio.3000791

**Published:** 2020-11-24

**Authors:** Rachel Lowe, Sadie J. Ryan, Roché Mahon, Cedric J. Van Meerbeeck, Adrian R. Trotman, Laura-Lee G. Boodram, Mercy J. Borbor-Cordova, Anna M. Stewart-Ibarra

**Affiliations:** 1 Centre on Climate Change and Planetary Health, London School of Hygiene & Tropical Medicine, London, United Kingdom; 2 Centre for Mathematical Modelling of Infectious Diseases, London School of Hygiene & Tropical Medicine, London, United Kingdom; 3 Department of Geography, University of Florida, Gainesville, Florida, United States of America; 4 Emerging Pathogens Institute, University of Florida, Gainesville, Florida, United States of America; 5 School of Life Sciences, University of KwaZulu-Natal, Durban, South Africa; 6 The Caribbean Institute for Meteorology and Hydrology, St. James, Barbados; 7 Caribbean Public Health Agency, Port of Spain, Trinidad & Tobago; 8 Facultad de Ingeniería Marítima y Ciencias del Mar, Escuela Superior Politécnica del Litoral (ESPOL), Guayaquil, Ecuador; 9 Inter-American Institute for Global Change Research, Montevideo, Department of Montevideo, Uruguay; University of York, UNITED KINGDOM

## Abstract

Small island developing states in the Caribbean are among the most vulnerable countries on the planet to climate variability and climate change. In the last 3 decades, the Caribbean region has undergone frequent and intense heat waves, storms, floods, and droughts. This has had a detrimental impact on population health and well-being, including an increase in infectious disease outbreaks. Recent advances in climate science have enhanced our ability to anticipate hydrometeorological hazards and associated public health challenges. Here, we discuss progress towards bridging the gap between climate science and public health decision-making in the Caribbean to build health system resilience to extreme climatic events. We focus on the development of climate services to help manage mosquito-transmitted disease epidemics. There are numerous areas of ongoing biological research aimed at better understanding the direct and indirect impacts of climate change on the transmission of mosquito-borne diseases. Here, we emphasise additional factors that affect our ability to operationalise this biological understanding. We highlight a lack of financial resources, technical expertise, data sharing, and formalised partnerships between climate and health communities as major limiting factors to developing sustainable climate services for health. Recommendations include investing in integrated climate, health and mosquito surveillance systems, building regional and local human resource capacities, and designing national and regional cross-sectoral policies and national action plans. This will contribute towards achieving the Sustainable Development Goals (SDGs) and maximising regional development partnerships and co-benefits for improved health and well-being in the Caribbean.

## Abstract 2

This Perspective article discusses progress towards bridging the gap between climate science and public health decision-making, highlighting the importance of multi-institutional and interdisciplinary collaborations to develop early warning systems for arboviral diseases in the Caribbean.

## Introduction

Frequent and intense extreme climatic events in the Caribbean, such as heat waves, Saharan dust incursions, hurricanes, floods, and droughts, have a detrimental impact on human health and development in the Caribbean region [1]. Adverse health outcomes include respiratory complications, heat-induced morbidity, outbreaks of vector- and water-borne diseases, injury, and loss of life [2]. The social and economic cost of these climate-sensitive health outcomes poses a disproportionately large burden to small island developing states, which have limited capacity to respond to the repeated onslaught of geophysical and hydrometeorological disasters. Recent advances in climate science are increasing our ability to anticipate and build resilience to climate-sensitive public health challenges [3]. The links between climate variations and epidemics of infectious disease are well established, with some studies demonstrating the potential for climate-based early warning systems for malaria and dengue [4–9]. Climate services for health is an emerging field of applied science. It brings together multisectoral professionals to cocreate tools and services that can help better understand and take advantage of the influence of weather and climate on health outcomes, such as epidemics of infectious disease [1,7,10]. Access to high-quality, tailored climate information can help health decision-makers better understand the influence of extreme weather, climate variability, and climate change on disease transmission and spread [11]. This combined with robust disease prediction frameworks can allow us to anticipate when, where, and who may be at greatest risk. When these decision support tools are effectively tailored to the needs of the public health sector, they can inform targeted interventions, promote the efficient use of human and financial resources, and reduce morbidity and mortality.

**Fig 1:**
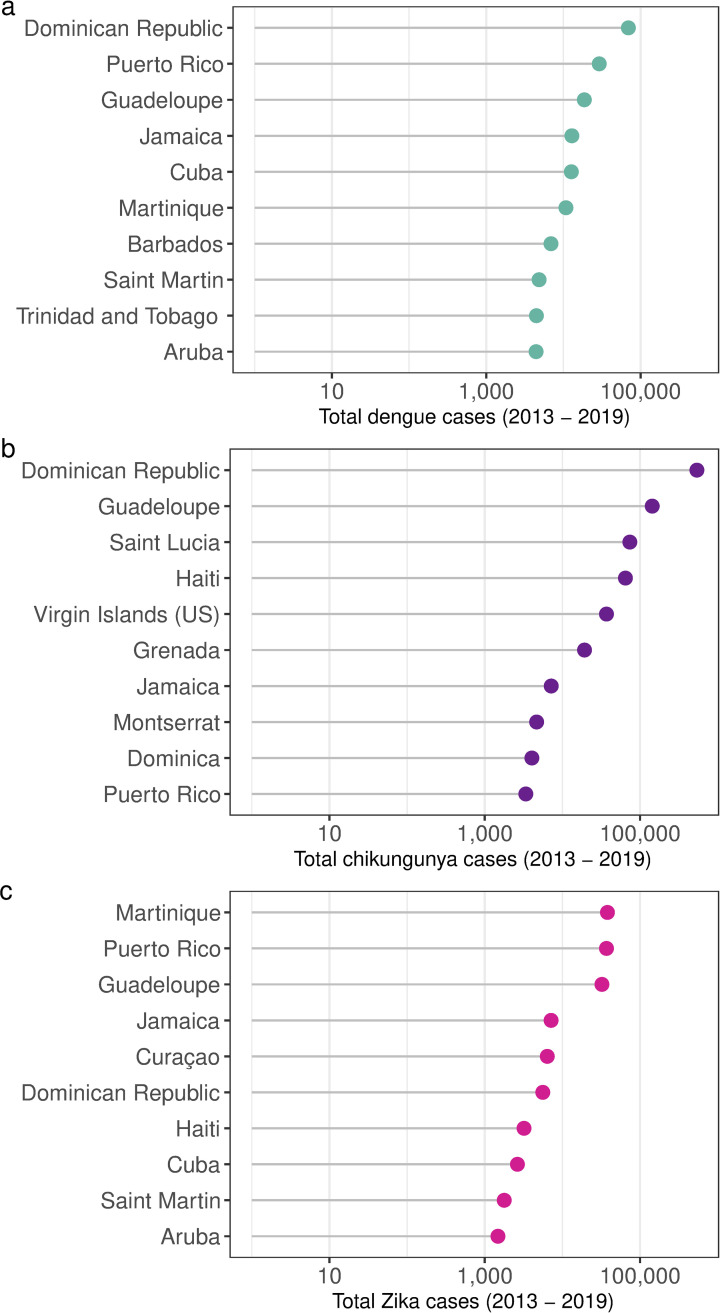
Total dengue, chikungunya, and Zika cases 2013 to 2019. Total cases of (a) dengue, (b) chikungunya, and (c) Zika between 2013 and 2019 per country. The 10 countries reporting the most cases in the Caribbean are shown for each disease. Note logarithmic scale. Source: PLISA Health Information Platform in the Americas [19].

Dengue is a viral infection transmitted to humans by female *Aedes* spp. mosquitoes. The global incidence of dengue has increased 30-fold in the last 50 years, and the disease is now endemic in more than 120 countries in the tropics and subtropics [12]. Prior to 2005, local transmission of chikungunya and Zika virus, which are transmitted to humans by the same mosquito species (*Aedes* spp.) as dengue, was limited to Africa and Asia. Since then, these viral diseases have spread globally, following the path of dengue [13]. Chikungunya emerged in the Caribbean in 2013, leading to more than 1 million cases in the region of the Americas within a year [14]. Zika reached the Americas in late 2014, resulting in the declaration of a Public Health Emergency of International Concern by the World Health Organization (WHO) due to links with neurologic complications in newborns and adults [15]. To date, 86 countries and territories have reported evidence of mosquito-transmitted Zika infection [16]. In 2019, the Americas experienced more than 3 million cases of dengue, the largest number ever reported [17]. In recent years, the Caribbean region has experienced an unprecedented crisis of co-occurring epidemics of febrile illness due to dengue, chikungunya, and Zika viruses [18]. Between 2013 and 2019, 186,050 cases of dengue, 911,842 cases of chikungunya, and 143,127 cases of Zika were reported in the Caribbean [19]. Fig 1 shows the 10 countries reporting the most cases of dengue, chikungunya, and Zika in the Caribbean between 2013 and 2019.

*Aedes aegypti* is the primary vector of dengue, chikungunya, and Zika viruses, a mostly domestic, urban mosquito that lays its eggs in water-bearing containers in and around the home. *Ae*. *aegypti* thrive in warm and humid conditions. Warm temperatures with an optimum peak of around 28.5°C increase the risk of *Ae*. *aegypti*–transmitted diseases by speeding up mosquito development rates, reproduction, survival, biting rates, and viral replication in the mosquito [20]. The effect of rainfall on dengue transmission is more complex, depending on the local social–ecological conditions and water management, which determines the type and abundance of larval habitat in the environment. Rainfall can increase mosquito population densities by increasing the availability of larval habitat in rain-filled containers out in the open. However, drought conditions that result in household water scarcity can also potentially increase larval habitat by increasing the number of water storage containers in and around the home.

In this perspective, we discuss the state of the art in climate services for health in the Caribbean region, with a focus on early warning systems for mosquito-transmitted diseases, such as dengue, chikungunya, and Zika. We highlight the importance of multi-institutional and transdisciplinary collaborations at national, regional, and international levels for research-to-operations-to-research (i.e., demand driven) innovation in the coproduction of climate services for health. These policy advances are of relevance to scientists and multisectoral decision-makers anticipating the impacts of climate extremes, variability, and climate change on health system capacity to respond to mosquito-borne disease outbreaks.

### Climate services in the Caribbean

There is a growing body of evidence on the impacts of climate extremes, variability, and climate change on human health in the Caribbean, including increases in acute asthma admission from Saharan dust clouds [21], increased dengue incidence during El Niño years [22], increase in the effect of high summer temperatures on mortality from stroke and cardiovascular diseases in Puerto Rico [23], and loss of life and injuries in Dominica from landslides caused by Hurricane Maria [24]. Despite this knowledge, small island developing states in the Caribbean have struggled to integrate climate services into national and health sector planning and practice [1]. Many countries in the region do not have sufficient institutional arrangements in place, or financial resources available, to stimulate collaboration, data sharing, research, or capacity building opportunities necessary to develop operational public health decision support tools and resources. In its role as the World Meteorological Organization (WMO) designated Regional Climate Centre (RCC) for the Caribbean, the Caribbean Institute for Meteorology & Hydrology (CIMH) leads the implementation of the Global Framework for Climate Services (GFCS) in the Caribbean. The GFCS is an international initiative led by the WMO, which acts as the guiding mechanism to support the development of climate services for key sectors of society. Since 2015, the CIMH has actively worked on an emerging, multipronged health–climate portfolio as part of its implementation of a regional multisectoral Early Warning Information Systems across Climate Timescales (EWISACTs) programme [1]. For the health sector, the CIMH, in collaboration with national and regional partners, such as Ministries of Health, National Meteorological and Hydrological Services, the Caribbean Public Health Agency (CARPHA), and the Pan American Health Organization (PAHO), aims to strengthen communication and partnership between climate and health actors and to promote the effective use of climate information within health policy, research, and practice.

Better management of mosquito-borne diseases is a top priority for Caribbean small island developing states given the high socioeconomic burden, suitable climatic conditions for the proliferation of *Aedes* mosquitoes, and widespread intra and extraregional travel in the Caribbean [2]. Thanks to the Building Regional Climate Capacity in the Caribbean (BRCCC) Programme, funded by the United States Agency for International Development (USAID) [25], the CIMH partnered with CARPHA, national Ministries of Health, and an international research team to support the development of regional arbovirus forecast models using climate information. This collaboration has aimed to codesign, codevelop, and codeliver health sector–driven climate early warning information to enhance the control and prevention of mosquito-borne disease outbreaks.

### Stakeholder needs and perceptions

A recent study of climate and health stakeholders in Barbados and Dominica [10] identified key strategies to promote climate services for public health decision-making. These included engaging senior leadership from the health sector to ensure that climate is high on the health agenda, formal collaboration agreements between climate and health sectors to facilitate a joint workplan, data sharing and modelling, national committees on climate and health, and joint spaces of dialogue, such as climate and health forums. Beyond the climate and health sectors, there is a web of institutional actors who should engage strategically to enhance arbovirus control and prevention, including disease risk management agencies, water and waste management companies, tourism, land planning, the Ministry of Environment, the Ministry of Agriculture, academia, and community organisations, to name a few [26]. Identifying priorities and gaps in concert would strengthen the partnership among the sectors, to enhance the development of climate services for health beyond the ministries and offices of public health [27]. Regional institutions should work in cooperation to build technical capacities and resilient communities across the region. The CIMH continues to actively strengthen its production network and user interface platforms to encourage stakeholders to share lessons and promote awareness of climate services based on user needs for all sectors, including health [28,29]. The Climate Investment Funds Pilot Programme For Climate Resilience (CIF/PPCR), which supports regional Inter-American Development Bank (IDB) projects, allows implementing institutions such as the University of the West Indies (UWI) in partnership with CARPHA and CIMH, and other regional/international teams to promote the development and implementation of climate-integrated disease surveillance systems in Caribbean countries, incorporating climatic, epidemiological, and entomological data. While multisectoral efforts provide a major impetus for reducing the burden of mosquito-borne diseases, the most successful response to early warnings of mosquito-borne disease is through community engagement.

### Early warning systems for mosquito-borne diseases

**Fig 2:**
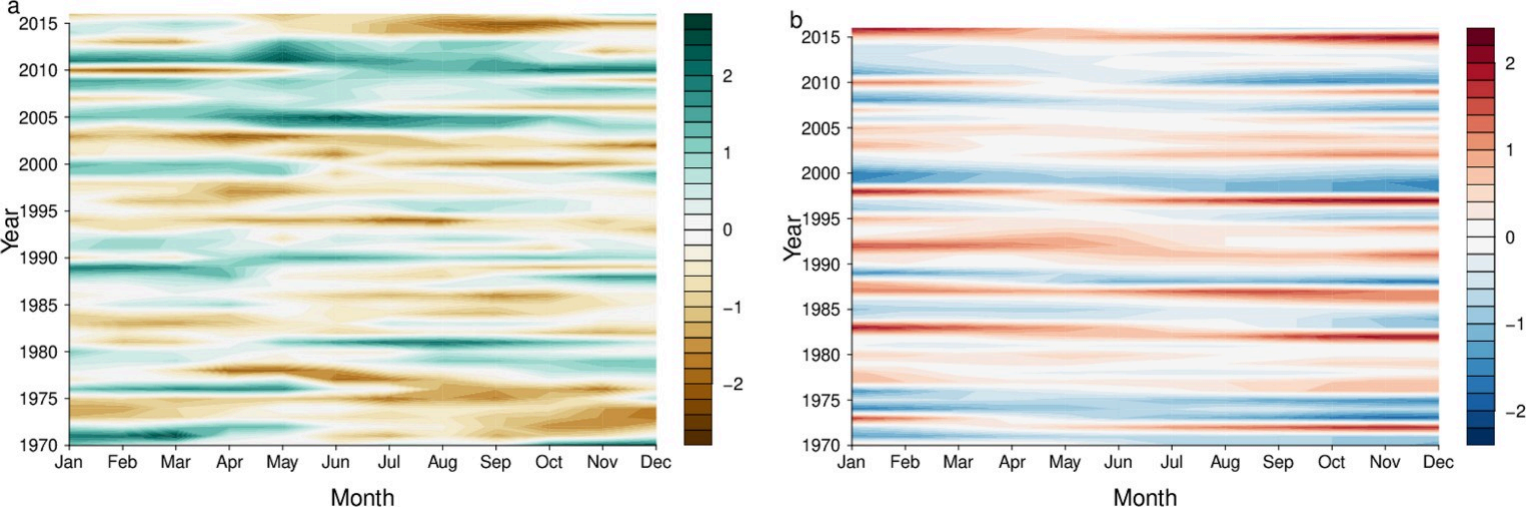
Annual cycle of the SPI in Barbados and the ONI 1970 to 2016. Annual cycle of (a) SPI (6-month timescale) (SPI-6) calculated using precipitation data from the CIMH synoptic weather station (elevation of 112 m) located in the western parish of St. James, Barbados. (b) ONI, defined as the 3-month running-mean sea surface temperature departures from average in the Niño 3.4 region (120–170°W, 5°S–5°N), from January 1970 to December 2016. Source: NOAA/NWS/CPC. Severe droughts (negative SPI-6 values; brown shading) tend to coincide with El Niño events (positive ONI values; red shading). CIMH, Caribbean Institute of Meteorology and Hydrology; ONI, Oceanic Niño Index; SPI, Standardised Precipitation Index.

The island of Barbados is among the 10 countries reporting the most cases of dengue in the Caribbean (Fig 1A). It is also among the 10 most water-stressed countries in the world, with water scarcity exacerbated during periods of drought [18]. In recent decades, Barbados has suffered from severe drought, which is often associated with the warm phase of the El Niño Southern Oscillation (Fig 2).

To mitigate the impacts of prolonged droughts, Barbados passed building regulations to mandate the construction of rainwater storage receptacles under large new buildings. However, these receptacles have the potential to become ideal larval habitat for *Ae*. *aegypti* mosquitoes if not properly covered and managed. This measure may have had the unintended consequence of increasing the overall risk of *Aedes*-transmitted diseases when rainfall is collected and stored. A link between temporary water storage and dengue risk has been recognised, but few studies have quantified the link or examined the impact of prolonged drought on dengue transmission.

To address this gap, Lowe and colleagues designed a coupled model framework to quantify the nonlinear and delayed impacts of climate factors, such as drought and extreme rainfall, on dengue transmission in Barbados from 1999 to 2016 [18]. The model is designed to disentangle the impacts of climatic drivers from socioeconomic risk factors, new viral introductions, immunological risk factors, and other unmeasured sources of variation. Key climate indicators included minimum temperature and the Standardised Precipitation Index (SPI) [30], an indicator used to monitor drought and extreme rainfall across the Caribbean. Drought conditions were found to positively influence dengue relative risk at long lead times of up to 5 months, while higher minimum temperatures and excess rainfall increased the risk at shorter lead times between 1 and 2 months. Therefore, periods of drought followed by a combination of warm and wet weather several months later could provide optimum conditions for imminent dengue outbreaks. The model was able to distinguish dengue outbreaks versus non-outbreaks in most years, although the dengue model performance in later years was likely compromised by the emergence of chikungunya and Zika in Barbados.

This modelling approach, which infers the risk of dengue outbreaks given the cumulative effect of climate variations in the preceding months, is currently being extended to other countries and tested as an operational early warning tool. The CIMH routinely produces seasonal forecasts of the SPI, minimum temperature, and other climate indicators, with a lead time of up to 3 months. By incorporating operational seasonal climate forecasts in the dengue prediction model, probabilistic dengue outlooks could be issued at least 3 months in advance of any given month and updated on a monthly basis, by incorporating new meteorological observations and shorter lead climate forecasts. One avenue for communicating dengue outlooks is through the quarterly Caribbean Health Climatic Bulletin, coproduced by the CIMH, PAHO and CARPHA [31]. The online Bulletin provides consensus-based expert statements of probable health outcomes based on CIMH’s monitoring and forecast products. Integration of quantitative probabilistic disease risk forecasts could help plan timely interventions to mitigate the impact of mosquito-borne disease epidemics, including purchasing of diagnostic and insecticide supplies, retraining of health professionals, realignment of vector control strategies, community mobilisation, and water storage container cleaning and maintenance during and after droughts. However, simply transmitting early warnings is not enough. It is essential for national health promotion personnel to work with local groups, such as civil society groups, community councils, and faith-based organisations, to design locally relevant community-based interventions in response to early warnings. Without thoughtful community involvement, it is very difficult to change behaviours and to mobilise an effective response to the warnings, however accurate the warnings may be. Ultimately, it is paramount that local authorities invest in longer-term solutions, including adequate infrastructure to secure provision of reliable piped water and ensure environmental hygiene for vulnerable communities.

### Capacity gaps, data management, and integrated surveillance systems

The development and effective mainstreaming of climate services for health, such as climate-driven dengue early warning systems, require parallel efforts to increase the capacity of the climate and health sectors in the region. National health stakeholders in Dominica and Barbados identified significant gaps in the availability of financial resources to implement an operational early warning system and a self-reported lack of technical expertise in statistics, data science, and geographic information systems (GIS) [10]. They also noted the lack of long-term health datasets and few local empirical studies on climate and health linkages, which limited their ability to make informed public health decisions. National meteorology and hydrology services also identified a lack of trained personnel and knowledge of health sector needs.

There is a critical need to train and retain a cohort of climate and health practitioners. In particular, the health sector requires technical training in how to analyse and interpret basic climate information and integrate this information into health surveillance and planning, whereas the climate sector requires more knowledge about the decision priorities and needs of the health sector. Further to this, resources must be allocated to reconciling data holdings across sectors. Historical records are often inconsistent in format and primarily paper based, while decentralised collection and storage complicates archiving or digitising. Country personnel trained in data management, from collection to data entry to quality control to data sharing, may be a limiting factor. Following recommendations from the Global Vector Control Response [26], vector and epidemiological surveillance systems should be integrated with climate information systems to allow for linkages across vector, disease, intervention, and climate data. This integrated system should also be homogenous and compatible between countries in the Caribbean. An important step towards improving climate-informed epidemic forecasting is through data and code sharing via the coordinated use of public repositories by multisector data providers. Following the example of Ecuador, Puerto Rico, and other countries that publish historic and real-time disease surveillance and climate data would promote the development of multi-institutional, multi-model ensemble approaches to improve the skill and utility of epidemic forecasts [32].

Capacity building needs are being addressed, in part, through the development of an online open access course on climate and health by a consortium of international experts led by WHO, PAHO, and WMO.

### Regional policy mechanisms

Regional action on (1) the SDGs agenda, (2) the climate actions proposed by Caribbean small island developing states under the Paris Climate Agreement, (3) the strategic priority areas outlined by the 10-year Caribbean Community (CARICOM) Caribbean Cooperation in Health (CCH) (2016 to 2025) initiative [33], and (4) the more recent PAHO-led Caribbean Action Plan on Health and Climate Change [34] offer an opportunity for synergies between the climate and health sectors to increase resilience for mosquito-borne diseases and other health impacts within the CARICOM. The co-development of climate services for mosquito-borne diseases contributes to promoting good health of communities (SDG3), making cities, towns, and communities more resilient (SDG 11), developing tools for reducing vulnerability to climate health impacts (SDG 13), and strengthening international collaboration through networking and sharing experience (SDG 17). The CARICOM experience provides evidence of the relevance of developing policy interactions across the SDGs and the existing integrated regional health–climate policy framework, to avoid policymakers and public health planners operating in silos [10].

At the national level, countries in the Caribbean are developing and implementing their National Development Plans, National Adaptation Plans for climate change, and disaster risk reduction strategies. The PAHO has also led efforts to develop Health National Adaptation Plans focused on climate resilient health systems [2]. Policy mechanisms to promote intersectoral and transdisciplinary work will enhance the effectives of these initiatives. For example, the climate services for health example presented in this perspective included participation of policy practitioners and experts from diverse disciplines. Moreover, the Nationally Determined Contributions (NDCs) include mitigation and adaptation measures that can generate co-benefits or additional benefits when nations act to reduce vulnerability and adapt to the impacts of climate change [35]. For example, adaptation efforts aimed at improving water management could also reduce the burden of mosquito-borne diseases.

## Summary

The Caribbean climate and health sectors are beginning to work together to attract the resources needed to increase local capacities to develop climate services for the health sector [10]. However, more work is needed to strengthen the partnership between climate information providers and health sector stakeholders to develop sustained climate services for mosquito-borne diseases in vulnerable Caribbean small island developing states. There are numerous areas of ongoing biological research aimed at better understanding the impact of climate variability and climate change on the transmission of mosquito-borne diseases. Recent studies have addressed key biological research questions, providing evidence on the linkages between climate variation and mosquito-borne disease outbreaks and demonstrating the benefits of effectively integrating climate information into public health decision-making processes. In line with international and regional calls for climate-smart health systems, successful projects, case studies, and tools should be replicated in similar settings to maximise their potential impact [10,18]. A demonstration of the benefits of climate services for mosquito-borne disease interventions in 1 country can also be used as a model for other climate-sensitive sectors (tourism, water supply, and disaster risk management) and other countries in the region to enhance preparedness for climate risks and health-promoting mitigation policies [35]. A major obstacle to developing sustainable climate services for health is a lack of financial resources, technical expertise, and data and knowledge sharing between sectors and disciplines. Investments should be made to more effectively link climate information to public health decision-making to reduce the impacts of climate variability and climate change and contribute to the health and livelihood of people and communities. National and regional policies aimed at achieving the SDGs present an opportunity for synergies and co-benefits for the well-being and development of Caribbean small island developing states.

## Abbreviations

BRCCC: Building Regional Climate Capacity in the Caribbean
CARICOM: Caribbean Community
CARPHA: Caribbean Public Health Agency
CCH: Caribbean Cooperation in Health
CIMH: Caribbean Institute for Meteorology and Hydrology
EWISACTs: Early Warning Information Systems across Climate Timescales
GFCS: Global Framework for Climate Services
GIS: geographic information systems
NDC: Nationally Determined Contribution
PAHO: Pan American Health Organization
PPCR: Pilot Programme for Climate Resilience
RCC: Regional Climate Centre
SDGs: Sustainable Development Goals
USAID: United States Agency for International Development
UWI: University of the West Indies
WHO: World Health Organization
WMO: World Meteorological Organization
